# Exploiting Necroptosis for Therapy of Acute Lymphoblastic Leukemia

**DOI:** 10.3389/fcell.2019.00040

**Published:** 2019-03-19

**Authors:** Caterina Mezzatesta, Beat C. Bornhauser

**Affiliations:** Department of Oncology and Children’s Research Center, University Children’s Hospital Zurich, Zurich, Switzerland

**Keywords:** necroptosis, leukemia, drug resistance, necroptotic compounds, apoptosis dysregulation

## Abstract

Escape from chemotherapy-induced apoptosis is a hallmark of drug resistance in cancer. The recent identification of alternative programmed cell death pathways opens up for possibilities to circumvent the apoptotic blockade in drug resistant cancer and eliminate malignant cells. Indeed, we have recently shown that programmed necrosis, termed necroptosis, could be triggered to induce cell death in a subgroup of primary acute lymphoblastic leukemia (ALL) including highly refractory relapsed cases. In this review we focus on molecular mechanisms that drive drug resistance in ALL of childhood and discuss the potential of necroptosis activation to eradicate resistant disease.

## Introduction

Acute leukemia is a hematological malignancy that perturbs the normal function of the hematopoietic system with fatal outcome if left untreated. Substantial improvement in the treatment of patients with childhood acute lymphoblastic leukemia (ALL) has been achieved over the last decades (Pui et al., 2015). Despite this success that is based on intensive chemotherapy protocols established in international collaborative studies (Schrappe et al., 2013), relapsed leukemia still ranks among the most common diagnoses of childhood malignancies, and survival rates of relapsed ALL remain low (Bhojwani and Pui, 2013). Thus, new treatment approaches have to be developed, in particular for relapsed ALL patients. In addition to immunotherapy, most current treatment approaches focus on targeting oncogenic lesions to induce cell death (Muschen, 2018). Enormous efforts over the recent years have identified and characterized the genomic lesions that occur in ALL (Mullighan et al., 2007; Fischer et al., 2015; Richter-Pechanska et al., 2018). Chromosomal translocations frequently affecting transcription factors combine with deletions in genes that regulate B- and T-cell development and mutations in genes that drive proliferation (e.g., CRLF2, RAS, ILR7, STAT5, Notch) (Mullighan et al., 2007; Fischer et al., 2015; Richter-Pechanska et al., 2018). The latter frequently occur at subclonal level. This heterogeneity and diversity of molecular lesions in ALL (Mullighan et al., 2007; Liu et al., 2017) has rendered the development of targeted therapies very challenging. In particular, chimeric translocations remain largely undruggable, and direct targeting of deletions is obviously not possible. Many of these alterations lead to reprogramming of hematopoietic differentiation and deregulation of molecular mechanisms that balance cell death and survival, providing the basis for poor response to chemotherapy and failure to undergo apoptosis. At the same time, this deregulation of signaling pathways also identifies nodes that could be targeted using small molecules and novel approaches. Among these, exploiting cell death mechanisms independent on classical apoptosis and caspases activation represents a particularly attractive alternative, with the potential to activate cell death responses under circumstances that prevent caspase-dependent cell death. Indeed, activation of necroptosis using the small molecule SMAC mimetic birinapant eliminated refractory leukemia cells in samples from highly resistant ALL patients (McComb et al., 2016). Corroborating these results, several compounds such as other SMAC mimetics or natural products are able to trigger the necroptotic pathway in leukemia but also in different carcinomas (Han et al., 2007; Fu et al., 2013; McCabe et al., 2014; Brumatti et al., 2016; Hannes et al., 2016; He et al., 2017; Safferthal et al., 2017). The possibility to develop and use drugs to induce necroptosis render this cell death mechanism very attractive for therapeutic approaches to eradicate malignant cells.

## Alteration Of Cell Death and Survival Signaling as Mechanisms of Drug Resistance in All

**FIGURE 1:**
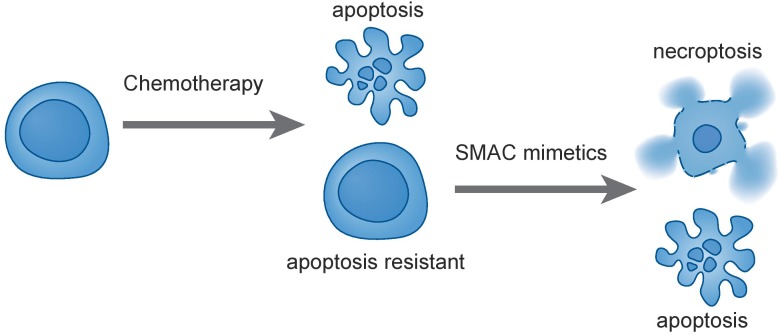
Treatment of leukemia cells with traditional chemotherapies can select for apoptotic-resistance. SMAC mimetics such as birinapant induce concurrent apoptosis and necroptosis in ALL and are thus active against apoptosis-resistant cells. This may contribute to eradication of resistant and refractory leukemia.

Comparison of ALL samples at diagnosis and relapse identified genomic and cytogenetic changes (Raimondi et al., 1993; Mullighan et al., 2008; Muschen, 2018; Richter-Pechanska et al., 2018) that are disease-driving and contribute to occurrence of relapse. Indeed, refractory ALL samples frequently present with secondary genetic alterations that arise from a minor subclone at diagnosis, which becomes predominant at relapse conferring drug resistance. Many of these alterations induce deregulation of pro- and anti-survival signaling pathways. Aberrant activation of the PI3K/AKT/mTOR axis is associated with poor clinical outcome in ALL, and its dysregulation can induce cell survival and resistance to cytotoxic drugs (Batista et al., 2011; Gomes et al., 2014; Khanna et al., 2018). Inhibition of this key pro-survival pathway, for instance using arsenic trioxide treatment, can resensitize steroid poor responder patients to glucocorticoids, key components of first-line ALL therapy. Arsenic trioxide increases protein levels of the BH3-only protein BAD, a pro-apoptotic member of BCL2 family and decreases the levels of the caspase inhibitor XIAP (Bornhauser et al., 2007). As shown in a case report of a refractory T-ALL patient, treatment with arsenic trioxide could induce complete remission without minimal residual disease (Wu et al., 2016). More direct inhibitors of this pathway, such as PI3K inhibitors or dual PI3K/mTOR inhibitors have shown promising activity in preclinical ALL models (Fruman et al., 2017). ALL refractory to glucocorticoids presented with high expression levels of the anti-apoptotic BCL2 family protein MCL1, due to a hyper activation of the PI3K/AKT/mTOR network (Wei et al., 2006), and specific MCL1 inhibitors are currently under evaluation for anti-leukemia activity (Ramsey et al., 2018). In refractory ALL, other possible dysregulation may more directly involve the apoptotic pathway and mitochondrial activity, which is controlled by the BCL2 family members. Indeed, correlation of drug resistance and alterations of BCL2 family proteins has been extensively described in leukemia (Letai et al., 2004; Campbell et al., 2010). Next to association of BCL2 family protein expression and drug resistance, these anti-apoptotic proteins also contribute to leukemogenesis. A transgenic mouse model showed a synergistic effect between BCL2 and c-MYC in malignant transformation of B-cells (Strasser et al., 1990). Moreover, an adaptation of the same mouse model demonstrated that presence of BCL-XL (anti-apoptotic BCL2 member) accelerates the development of MYC-driven leukemia (Swanson et al., 2004). Increased leukemia development was observed also in Eμ-Myc transgenic mice upon genetic disruption of one BIM (BCL2 pro-apoptotic protein) allele (Egle et al., 2004). Thus, dysregulation of pro- or anti-apoptotic BCL2 proteins can support malignant cell maintenance and survival also once the tumor is established. Recently developed diagnostic procedures with functional analysis of BCL2 family protein dependence using BH3 profiling (Ryan and Letai, 2013; Ryan et al., 2016; Touzeau et al., 2016) can be used to predict chemotherapeutic sensitivity in several cancer types (Ni Chonghaile et al., 2011). It has become clear from these approaches that a subset of ALL cases heavily depend on specific BCL2 family members. In order to target the interaction between pro- and anti-apoptotic BCL2 proteins in cancer, a new class of compounds, the BH3-mimetics, has been developed. In particular the BCL2-specific BH3-mimetic venetoclax (ABT-199) has shown high activity *ex vivo* and *in vivo* in a subset of B-cell precursor ALL (Fischer et al., 2015) and in some T-cell leukemia samples (Chonghaile et al., 2014; Peirs et al., 2014; Frismantas et al., 2017). Moreover, venetoclax has shown promising results also in clinical trials for other hematologic malignancies (Konopleva et al., 2016). However, high expression of MCL1 (Choudhary et al., 2015) or low expression ratio of BCL2 vs. BCL-XL may underlie a potential resistance to venetoclax. To overcome this, it is possible to combine MCL1 inhibitors with BCL2 inhibitors, which was shown to have a synergistic effect in preclinical studies (Leverson et al., 2015). While representing an important factor for drug resistance, dysregulation of BCL2 proteins is not the only cause for apoptotic rescue in malignant cells. Alterations in genes that drive metabolism have also been described to underlie drug resistance in ALL. Mutations in the nucleotidase NT5C that are recurrent in T-ALL (Tzoneva et al., 2013, 2018) may confer resistance to mercaptopurine, a key element in ALL therapy, representing a typical example of gain-of-function mutations that are difficult to target, which is in addition also associated with occurrence of relapse. Recent discoveries have highlighted the occurrence of the deletion of the B-cell transcription factor IKZF1 together with CDKN2A, CDKN2B, PAX5, or PAR1 to identify a subgroup of B-cell precursor ALL patients with exceedingly bad outcome (Stanulla et al., 2018). We are only at the beginning of understanding the consequences of such deletions on drug resistance. In addition to drive B-cell development, IKZF1 controls a metabolic program that includes regulation of responses to steroids (Marke et al., 2016; Chan et al., 2017), and its loss may be directly linked to steroid resistance. Next to metabolic alterations, a second group of pro-survival proteins, the inhibitor of apoptosis proteins (IAPs), are frequently highly expressed in leukemia (Tamm et al., 2004; Hundsdoerfer et al., 2010) and constitute relevant targets for intervention. The pro-survival activity of cIAP1/2 is linked with their ubiquitination activity and the ability to interact with and promote the survival activity of receptor-interacting protein kinase 1 (RIPK1) (Peltzer et al., 2016; Lalaoui and Vaux, 2018). Ubiquitination of RIPK1 enables its Nuclear Factor kappa B (NF-kB) activating potential, supporting survival also in cancer cells (Bertrand et al., 2008; Varfolomeev et al., 2008). Small molecules SMAC mimetics can target and inhibit the cIAPs, which induces deubiquitination of RIPK1 in the TNF receptor 1 (TNFR1) complex and subsequent activation of RIPK1-dependent death. These agents have shown anti-cancer activity in different solid tumor cell line models (Fulda, 2015). Moreover, primary ALL and acute myeloid leukemia (AML) samples undergo RIPK1-dependent death upon SMAC mimetics treatment (Brumatti et al., 2016; Lalaoui et al., 2016; McComb et al., 2016; Richmond et al., 2016). The tumor suppressor role of RIPK3 for AML development in mice (Hockendorf et al., 2016) further underscores the importance of this pathway in hematological malignancies. Interestingly, treatment with SMAC mimetics induced RIPK1-dependent concurrent apoptosis and necroptosis in primary ALL samples, both *in vitro* and *in vivo* in the xenograft model (McComb et al., 2016). The high anti-leukemic activity of SMAC mimetics is thus based on their potential to trigger necroptosis, to eradicate also refractory ALL cells that are unable to mount an apoptotic response (Figure 1). To further characterize and understand the potential of necroptosis activation for anti-leukemia therapy, it will be important to develop biomarkers that brand a response and to determine strategies to identify those patients who may benefit from such an approach.

## Activation of Necroptosis as Anti-Leukemia Therapy

**FIGURE 2:**
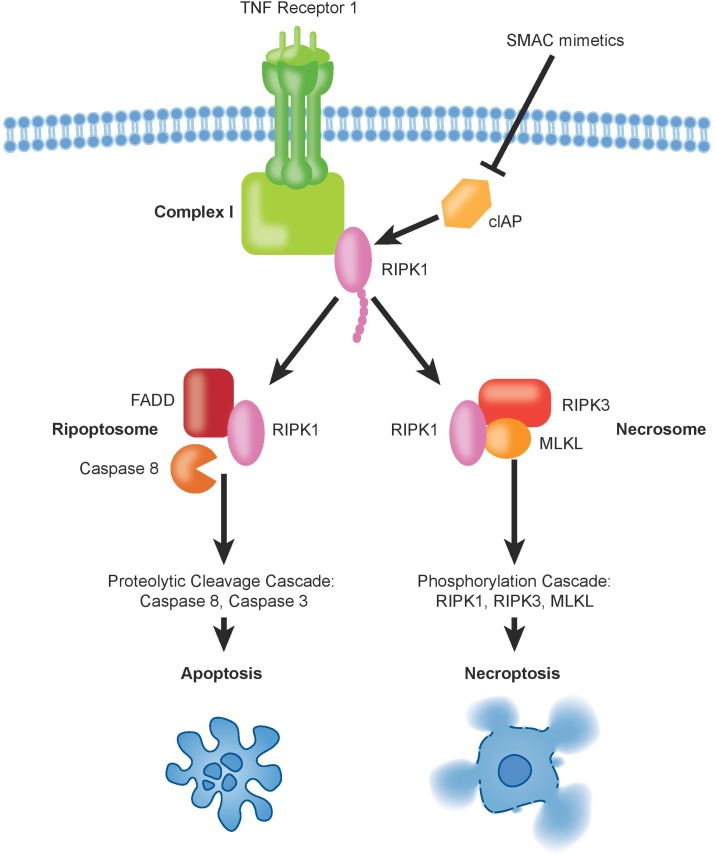
The binding of TNFα to TNFR1 induces the formation of complex-I, which contains also ubiquitinated RIPK1. Deubiquitination of RIPK1, upon inhibition of cIAPs by SMAC mimetics, can trigger formation of pro-death signaling complexes, either via apoptosis and/or necroptosis.

Despite its relatively recent description (Degterev et al., 2005), necroptosis ranks among the best described non-apoptotic and caspase-independent forms of cell death. It is a caspase-independent cell death mechanism, which presents necrotic features that are highly regulated (Wang et al., 2018). The signal transduction steps that govern necroptosis induce initiation and execution of this cell death pathway controlled by the RIP Kinases, ending with cell swelling and rupture of the cellular membrane, leading to the release of cellular content into the extracellular space (Kaczmarek et al., 2013). The main regulating players of this programmed cell death are RIPK1, RIPK3, and the mixed lineage kinase domain-like protein (MLKL) (Vanden Berghe et al., 2014). Experimentally, necroptosis is frequently triggered by exogenous tumor necrosis factor alpha (TNFα) in combination with pharmacological caspase inhibition using, e.g., zVAD, QVD, or emricasan. Other death receptors that can activate necroptosis in presence of their respective ligands include FAS (also known as CD95 or APO-1), DR3, TRAILR1, TRAILR2, and DR6 (Wilson et al., 2009). Mechanistically, TNFα binding induces oligomerization of TNFR1 and the formation of complex-I at the plasma membrane. Complex-I is a multiprotein complex that includes TNFRI, TNFR-associated death domain protein (TRADD), TNFR-associated factor-2 and 5 (TRAF2/TRAF5), the cIAPs1/2 and RIPK1 (Vanden Berghe et al., 2014). At this level, cell fate decisions are taken, with RIPK1 having multiple functions. Depending on its post-translational modifications, in particular ubiquitination status, RIPK1 controls cell survival or can activate cell death trough apoptosis and necroptosis. Poly-ubiquitination of RIPK1 driven by cIAPs1/2 and LUBAC triggers survival through NF-kB signaling, which leads to mitogen-activated protein kinase (MAPK) activation (Pasparakis and Vandenabeele, 2015). Simultaneously, ubiquitination of RIPK1 prevents necroptosis and RIPK1-dependent apoptosis activation. Deubiquitination of RIPK1 can induce the formation of the cytosolic complex-IIb, which comes in two different flavors. Under caspase-8 proficient conditions, deubiquitination of RIPK1 leads to formation of the ripoptosome leading to apoptosis through caspase-8 dependent mechanisms, while the necrosome is formed if caspase-8 is non-active (Wegner et al., 2017) (Figure 2). In the necrosome, RIPK1 associates with and phosphorylates RIPK3 leading to the oligomerization and translocation of MLKL to the plasma membrane (Zhao et al., 2012; Huang et al., 2017). It is worth noting that in particular the ripoptosome is fairly short lived and can usually only be detected under experimental caspase blockade using zVAD. The deubiquitination of RIPK1 may occur through activity of the deubiquitinases CYLD and A20 (Wright et al., 2007; Bonapace et al., 2010; Wegner et al., 2017) or through depletion of cIAP1/2 by SMAC mimetics treatment. To guide decisions between RIPK1-dependent apoptosis or necroptosis, autophagy genes were shown to play an important scaffolding role (Goodall et al., 2016). MLKL can be considered the executor of necroptosis as it induces formation of pores on the plasma membrane, which becomes permeable releasing damage-associated molecular patterns (DAMPs), thus ending into necroptosis (Dondelinger et al., 2014; Wang et al., 2014; Xia et al., 2016). The identification of RIPK1-dependent necroptosis to underlie the extraordinary sensitivity to SMAC mimetics in a subgroup of pediatric ALL represents an example in which experimental inhibition of caspase-8 is not required. Rather, we hypothesize that this may be due to the existence of specific but varying RIPK1-associated protein complexes within the cells. We could not identify any association of protein expression of either caspase-8, RIPK3, MLKL, cIAP1/2, or RIPK1 with sensitivity to SMAC mimetics in ALL (McComb et al., 2016), suggesting that the regulation and sensitivity will be more complicated than mere expression levels. Interestingly, our own data (McComb et al., 2016) demonstrated a TNFα-independent effect of SMAC mimetics, suggesting that auto- or para-crine regulation of TNFα by RIPK1 does not seem to play a major role for sensitivity. Comparative gene expression analyses suggest association of the Philadelphia-like ALL subgroup with sensitivity to SMAC mimetics, with TNFR1 expression correlating with response, while cFLIP did not appear amongst the most highly regulated genes (Richmond et al., 2016). Mutations in caspase-8 or epigenetic silencing has not been described in ALL so far (Mullighan et al., 2007; Liu et al., 2017), indicating that the underlying molecular mechanisms that determine sensitivity will be more complex than anticipated.

## Potential of Necroptosis-Inducing Compounds in All

Triggering necroptotic cell death should be considered as a new therapeutic approach in cancer treatment in order to eradicate malignant cells that are refractory to apoptotic drugs. Several agents, including natural and targeted compounds, have been shown to induce necroptosis in ALL, frequently also in combinatorial approaches. In particular combination of SMAC mimetics with the steroid dexamethasone (Rohde et al., 2017) and with demethylating agents (DAC) (Gerges et al., 2016), as well as inhibition of NF-kB (Meng et al., 2010) activate necroptosis in ALL cells, while hypertonicity enhanced activity of SMAC mimetics by combination of apoptosis and necroptosis (Bittner et al., 2017). The best well-known drugs that can induce necroptotic cell death are indeed the SMAC mimetics combined with caspase-8 inhibition (McCabe et al., 2014; Brumatti et al., 2016; Hannes et al., 2016). This type of treatment can push the cells to necroptosis due to inhibition of cIAPs, thereby inhibiting the pro-survival function of RIPK1, and on the other hand caspase inhibition confers a block in apoptosis. Interestingly, we have observed that refractory ALL samples could undergo necroptosis cell death also in absence of caspases inhibition upon the SMAC mimetic compound birinapant as single agent (McComb et al., 2016). Several SMAC mimetic compounds are already in phases I or II of clinical trials to treat leukemia and solid tumors (NCT02098161, NCT01188499, NCT01486784). It will be interesting to see if necroptosis contributes to a potential anti-tumor effect in these trials. Still, the most promising anti-tumor activity of SMAC mimetics may be achieved if combined with other anti-cancer agents. For instance, the SMAC mimetic compound BV6 synergizes with DAC, cytarabine, or HDAC inhibitors in acute myeloid leukemia (AML) (Steinhart et al., 2013; Chromik et al., 2014; Steinwascher et al., 2015). This activity required necroptosis for full efficacy. Antagonism of cIAPs may boost both innate and adaptive immune responses and increase tumor cell killing (Beug et al., 2017; Dougan and Dougan, 2018; Michie et al., 2019). In addition to SMAC mimetics, other agents are able to trigger a necroptosis response. Activation of necroptosis using drugs as 5-fluorouracil or staurosporine (Dunai et al., 2012; Grassilli et al., 2013; Oliver Metzig et al., 2016), again if caspases are inhibited, showed high anti-cancer potential. Moreover, necroptosis could be activated by shikonin, a natural compound derived from a plant extract, in leukemia and in multiple myeloma (Han et al., 2007; Wada et al., 2015). This compound and other analogs may overcome drug resistance due to expression of MRP1, BCRP1, P-glycoprotein, BCL2 and BCL-XL through necroptotic cell death (Han et al., 2007; Xuan and Hu, 2009). Furthermore, necroptosis has been described in some cases to depend on autophagy. In fact, the pan-BCL2 inhibitor obatoclax triggered autophagy-dependent necroptosis, thus restoring the response to the glucocorticoid dexamethasone in steroid-resistant ALL (Bonapace et al., 2010). Moreover, bypassing chemoresistance through autophagy-mediated necroptosis is possible upon chalcone treatment or using the tyrosine kinase inhibitor sorafenib (He et al., 2014; Kharaziha et al., 2015). One important aspect to be taken into account when considering necroptosis activation in cancer therapy is its potential immunogenicity. Disruption of the cellular membrane and release of DAMPs may activate immune responses that potentially can also act on the malignant cells. Indeed, vaccination with necroptotic cancer cells induces an adaptive immune response through cytotoxic CD8a+ T cells *in vivo*, which mediates efficient anti-tumor immunity (Aaes et al., 2016). Sometimes though, the release of DAMPs may not be sufficient for CD8+ T cell cross-priming, and RIPK1 signaling and activation of NF-κB within dying cells is in addition required to boost the response (Yatim et al., 2015). The question to what extent activation of necroptosis in ALL in particular, but also in other hematological malignancies such as AML (Brumatti et al., 2016) is immunogenic remains open. Some data from solid tumors suggest that necroptosis does not necessarily always have to be pro-inflammatory and immunogenic (Brouckaert et al., 2004; Lohmann et al., 2009). Still, while TNFα-induced necroptosis may even shut down inflammatory responses (Kearney et al., 2015), data with respect to cytokine release and inflammatory responses on necroptosis induced by SMAC mimetics are lacking, in particular also in the context of refractory ALL. Clearly, susceptibility to necroptosis-mediated cell death does represent a specific vulnerability of lymphoid cells, even without necessity to experimentally inhibit caspases. In the future, potential immunogenicity and inflammatory responses of necroptosis induction will have to be investigated carefully, in order to evaluate the therapeutic anti-leukemia potential of necroptosis induction.

## Author Contributions

All authors listed have made a substantial, direct and intellectual contribution to the work, and approved it for publication.

## Conflict of Interest Statement

The authors declare that the research was conducted in the absence of any commercial or financial relationships that could be construed as a potential conflict of interest.
